# Improving pressure injury risk assessment using real‐world data from skilled nursing facilities: A cohort study

**DOI:** 10.1111/iwj.70000

**Published:** 2024-07-12

**Authors:** Matthew Oliver Wynn, Lucas Goldstone, Rishabh Gupta, Justin Allport, Robert D. J. Fraser

**Affiliations:** ^1^ School of Health and Society University of Salford Salford UK; ^2^ Swift Medical Inc Toronto Ontario Canada; ^3^ Arthur Labatt Family School of Nursing Western University London Ontario Canada

**Keywords:** assessment, Braden, pressure injury, risk, survival model, time‐series

## Abstract

This study aimed to improve the predictive accuracy of the Braden assessment for pressure injury risk in skilled nursing facilities (SNFs) by incorporating real‐world data and training a survival model. A comprehensive analysis of 126 384 SNF stays and 62 253 in‐house pressure injuries was conducted using a large calibrated wound database. This study employed a time‐varying Cox Proportional Hazards model, focusing on variations in Braden scores, demographic data and the history of pressure injuries. Feature selection was executed through a forward‐backward process to identify significant predictive factors. The study found that sensory and moisture Braden subscores were minimally contributive and were consequently discarded. The most significant predictors of increased pressure injury risk were identified as a recent (within 21 days) decrease in Braden score, low subscores in nutrition, friction and activity, and a history of pressure injuries. The model demonstrated a 10.4% increase in predictive accuracy compared with traditional Braden scores, indicating a significant improvement. The study suggests that disaggregating Braden scores and incorporating detailed wound histories and demographic data can substantially enhance the accuracy of pressure injury risk assessments in SNFs. This approach aligns with the evolving trend towards more personalized and detailed patient care. These findings propose a new direction in pressure injury risk assessment, potentially leading to more effective and individualized care strategies in SNFs. The study highlights the value of large‐scale data in wound care, suggesting its potential to enhance quantitative approaches for pressure injury risk assessment and supporting more accurate, data‐driven clinical decision‐making.

## 1 INTRODUCTION

Pressure injuries (PIs), also known as pressure ulcers, are localized damage to the skin and/or underlying tissue that usually occurs over a bony prominence as a result of pressure, or pressure in combination with shear.^1^ PIs commonly occur in individuals who are unable to change positions independently such as those with acute conditions, are critically ill or have long‐term immobility. The prevalence of PIs is a significant concern, particularly in skilled nursing facilities (SNFs) where patients are often at an elevated risk due to various factors including limited mobility, age and chronic conditions putting them at similar levels of risk to hospitalized patients.^2^ Although the exact global economic impacts of these injuries are challenging to determine due to variations in data collection both regionally and globally, the burden of PI is high and likely to increase due to global ageing.^3^

Current risk assessment tools including the Norton,^4^ Braden^5^ and Waterlow^6^ tools have been so far limited by low sensitivity (ability to correctly identify at‐risk patients). They are also limited by their lack of consideration of temporality, with scores reflecting the patient risk at only one point in time and an assumption of linearity to risk factors, with scores being calculated by adding up risk factors and the subjective nature of some scale elements.^7^ These limitations reduce the predictive power and consequently the potential clinical value of such quantitative risk assessment tools. This is reflected in the most recent Cochrane review, which indicated that the use of pressure injury risk assessment tools (PIRAT), specifically the Braden and Ramstadius tools, had no clear relation to PI incidence.^8^ These limitations have resulted in some organizations removing quantitative risk assessment for PIs from policy and guidance. Most notably, in the UK, the National Wound Care Strategy Program (NWCSP) now advocate use of the Pressure Ulcer Risk Primary or Secondary Evaluation Tool (PURPOSE‐T) risk assessment tool.^9^ This tool makes no attempt to quantify risk and instead relies solely on the clinical judgement of practitioners. Whilst this is an understandable response to the demonstrable limitations of older risk assessment tools, there are also limitations to this approach. Not least that effective clinical judgement can only be exercised with the benefit of robust clinical research evidence, which is currently lacking; in addition, evidence indicating the link between structured risk assessment, clinical judgement and implementation and evaluation of preventative measures is also currently poor.^10^

Improving the assessment of PI risk in SNFs is crucial to better allocate resources, provide targeted care and ultimately prevent the occurrence of PIs. With the advent of electronic health records (EHRs), smartphone technology and the consequent increasing availability of large healthcare datasets, there is a growing opportunity to leverage real‐world data to enhance risk assessment tools, like the Braden Scale. By analysing detailed patient data from SNFs, including demographic information, medical history and specific care interventions, it is possible to develop more accurate and tailored risk prediction models. Such models may help healthcare providers identify patients at high risk for PIs more effectively and implement preventative measures in a timely manner. Work has already been undertaken in this area to develop more granular insights into the relative significance of risk factors included in existing PIRAT. For example, a recent study utilizing electronic patient data in China indicated that five out of 11 risk factors included in the Waterlow score were insignificant in relation to the development of PI.^11^ This study also noted that the Waterlow score failed to consider other risk factors which have been identified as significant risk factors (e.g., haematological, oxygenation and perfusion) for which data may be available in electronic record systems. Machine learning (ML) algorithms also offer promise in developing more dynamic and accurate risk prediction models by analysing comprehensive patient data from SNFs. Such models can facilitate timely and targeted preventive measures by identifying high‐risk patients more effectively. This potential is noted in a recent systematic review by Barghouthi et al.,^12^ which reported the efficacy of ML algorithms in early risk identification and prediction of PIs in hospitalized adult patients. Their review underscores the shift towards leveraging big data and algorithmic approaches to improve upon traditional, often subjective risk assessment tools.^12^

Our study sought to build on the existing literature, leveraging data available from the Swift database to train a survival model to improve the accuracy of quantitative risk assessment using the Braden tool risk scale factors as a foundation. Survival models are used in biomedical research to analyse the time until an event of interest occurs, such as the development of a pressure ulcer.^13^ These models accommodate censored data, where the outcome event (e.g., PI occurrence) may not be observed for all study subjects within the study period. This approach allows for the estimation of survival functions and the examination of how different factors influence the time to event. By incorporating time‐to‐event data, survival models can enhance understanding of the dynamics of PI risk over time, offering a more nuanced view that accounts for the progression of risk, which is particularly valuable in clinical settings that may benefit from timely risk‐based alerts to implement timely preventive measures.

The objective of this study was to improve Braden assessment by training a survival model using a large database of patient stays in SNFs.

## 2 METHODS

### 2.1 Data source and collection

The study utilized a large, scientifically calibrated (size and colour calibrated images using a standardized fiducial), Artificial Intelligence–based Wound Care Solution (AI‐WCS) dataset. The AI‐WCS captures wound images calibrated for size (lengths, width, depth) and colour and patient risk scores (e.g., Braden, Norton, Inlows). Information is securely captured within the application and encrypted in transit to secure cloud‐based dashboards or integrated electronic medical records (EMR).

Data used in this study were de‐identified using privacy requirements under the Health Insurance Portability and Accountability Act (HIPAA) of the US and the Personal Health Information Protection Action (PHIPA) in Canada. The Safe Harbour process was used to remove identifying information, and additional processes are in place to monitor the risk of re‐identification. For example, image data were screened by algorithms for text information (e.g., tattoos, patient labels) and facial recognition that excluded data to reduce risk of re‐identification further. Ethics review was completed with Pearl IRB, which provided an exception (2023‐0100) and the University of Salford ethics committee. Retrospective review of the de‐identified data did not result in changes to patient care or impact care provided. Swift Medical endorses the European Medical Association Declaration of Helsinki for human subject research.

SNFs participating in the data de‐identification process were included in this study. The data set allows de‐identified patient characteristics (e.g., age grouping in decades rather than date of birth) to be analysed and included Braden Scale's risk scores, wound evaluations and demographic data. Data provided by participating organizations between April 2016 and October 2022 were included in this study. The Braden scale was used in this study due to its recommendation within NPIAP (2019) guidance and wide adoption within US SNF.

The Braden tool is used in healthcare, particularly in nursing homes (NHs), to assess the risk of PI. The Braden Scale has reportedly greater reliability and validity, surpassing other scales such as Norton and Waterlow in sensitivity and specificity.^14^ However, it still has limited clinimetric value in relation to PI prediction overall.^8^ This, in addition to its popularity among SNF, justifies its use as the basis for development of a new PI risk assessment process. The Braden scale comprises six subscales: mobility, activity, sensory perception, nutrition, friction/shear and moisture. These subscales assess an individual's risk factors for PI, with lower total scores indicating a greater risk.^14^

### 2.2 Study design

Time‐series data of patient (period between an admission and a discharge) in SNFs were analysed. This included Admission, Discharge, Transfer (ADT) and data indicating what patients had existing or acquired wounds in the participating SNFs.

### 2.3 Data analysis

Patient stays were split into 10 folds for cross‐validation purposes, and each fold was split into training (90%) and testing (10%) datasets. The study employed a time‐varying Cox Proportional Hazards model to analyse the risk of developing PI, with the first documentation of an in‐house PI as the event of interest. This model allows us to calculate an interpretable risk score that varies over time using the feature coefficients from the fitted model.

### 2.4 Feature selection

A forward‐backward selection process was applied to choose relevant data attributes, whereby univariate models were first fit for each feature and compared with a null model using a likelihood ratio test. Features were included in a full (multivariate) model if they significantly improved goodness‐of‐fit compared with a null model (*p* < 0.05). In the backwards selection step, single features were dropped from the full model iteratively, and the reduced model's performance was compared with the full model using the Akaike information criterion (AIC). Features were removed from the final model if their omission decreased AIC. The study focused on Braden subscores, including score increase and decreases in consecutive assessments, patient age and sex, and the recent history of PIs.

### 2.5 Outcome measures

To assess the effectiveness of the improved risk scores, scores from the model's coefficients were compared with Braden scores using concordance indices and time‐dependent discrimination accuracy was compared using the formula AUC^I/D^ t.^15^ The relative importance of each attribute was estimated by comparing log likelihoods of the full model and a reduced model fitted without the feature of interest.

The dataset was large and diverse, sourced from multiple SNF across the US and Canada, reducing the risk of bias in participant selection. Feature selection was conducted using a forward‐backward method, discarding irrelevant data attributes and minimizing the chance of overfitting or bias towards insignificant factors. The use of a time‐varying Cox Proportional Hazards model allowed for a more dynamic and accurate representation of risk factors over time. Additionally, the dataset was balanced to include a similar number of patients with and without PIs, helping to mitigate selection bias. Finally, comparing the new model's risk scores with traditional Braden scores using concordance indices provided a comprehensive evaluation against established methods, enhancing the study's reliability.

## 3 RESULTS

The patient data in the study included a large cohort from SNF in the US. The study analysed data from 126 384 SNF stays across 3063 facilities and involving 102 170 patients. The demographic breakdown of the patients was 42% male and 58% female, with a median age range of 80–90 years. This diverse group provided a comprehensive data set for analysing the risk and occurrence of in‐house PI in SNFs. These patients were the subjects of a total of 791 235 Braden assessments (number of assessments per patient: mean = 7.74, median = 5). Over the course of the study, 42 321 patients developed a PI in‐house. Of those, 25.5% of patients (*N* = 10 803) developed more than one PI in‐house throughout their stay. The demographics of the study sample can be seen in Table 1.

**TABLE 1:** Sample demographics.

Sex	Age	Count	Sex	Age	Count
Female	0 to 9	9	Male	0 to 9	7
	10 to 19	12		10 to 19	25
	20 to 29	93		20 to 29	148
	30 to 39	293		30 to 39	426
	40 to 49	690		40 to 49	910
	50 to 59	2463		50 to 59	2839
	60 to 69	7330		60 to 69	7595
	70 to 79	14 645		70 to 79	12 258
	80 to 89	31 913		80 to 89	18 118
	90+	1599		90+	797
Total		59 047		Total	43 123

**FIGURE 1:**
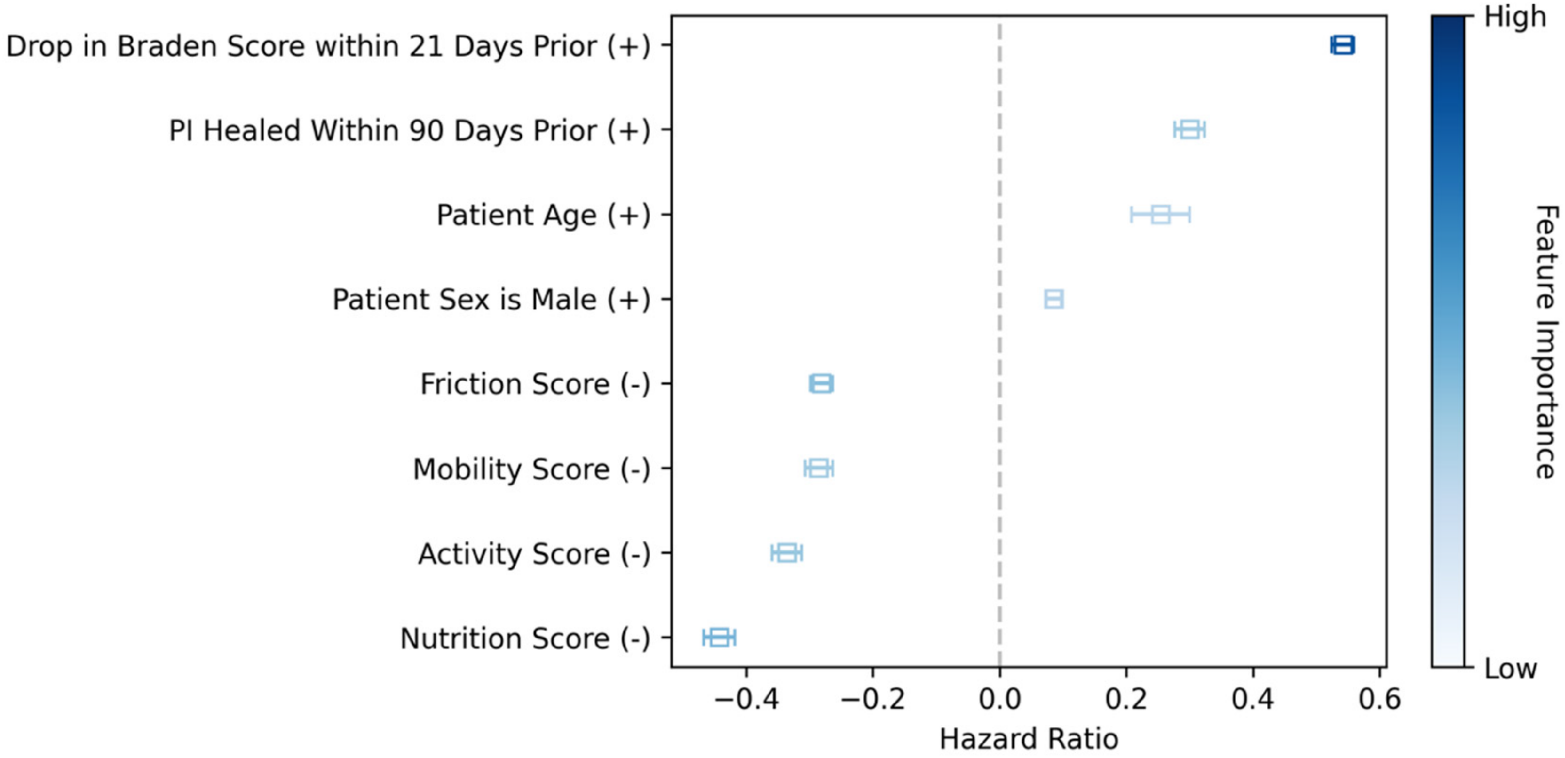
Hazard ratios for the features included in the model. Positive hazard ratios are associated with increased risk. Negative ratios are associated with decreased risk.

**FIGURE 2:**
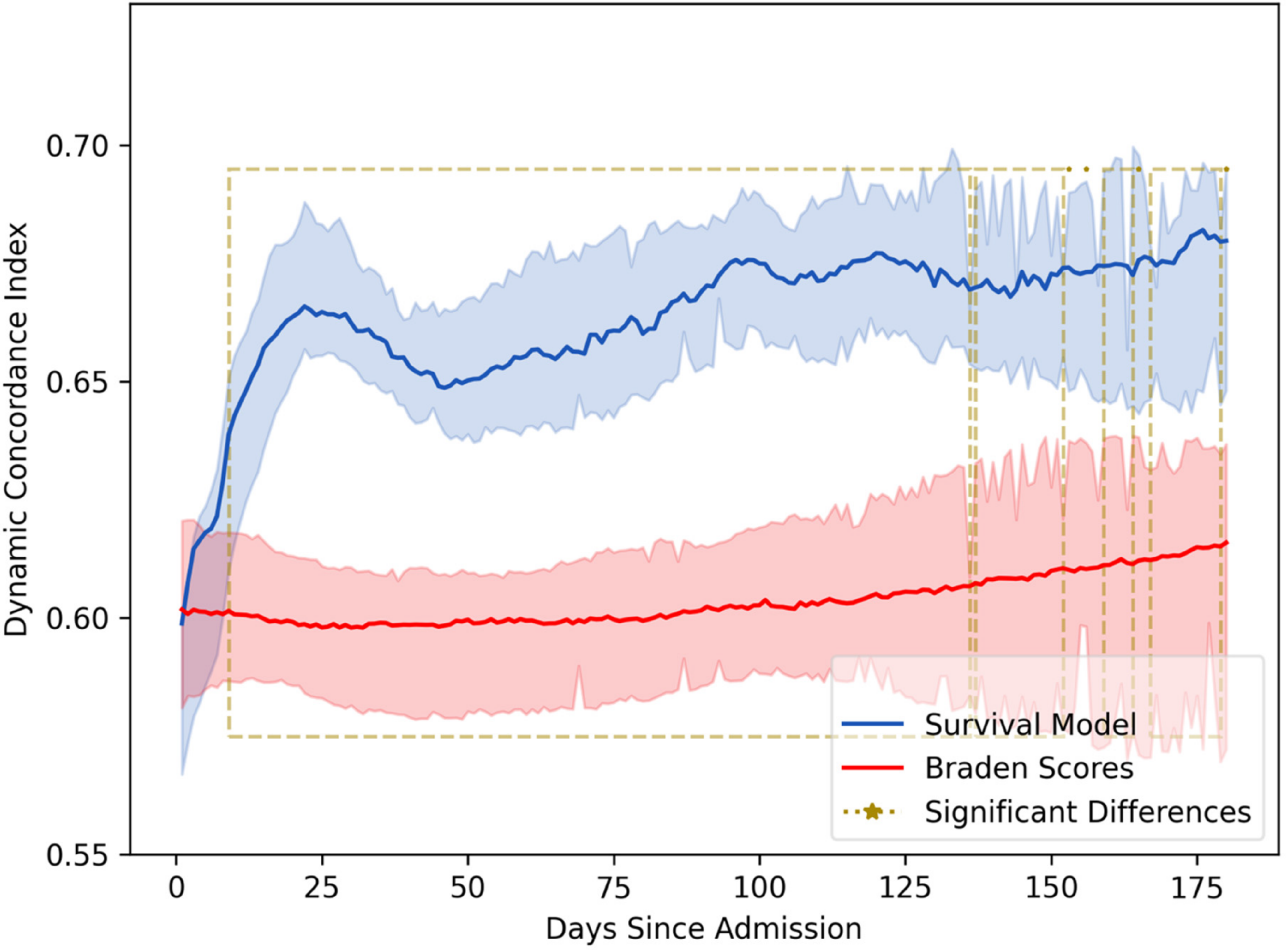
Global concordance indices of our survival model versus Braden across 10 training and testing splits of our dataset (blue trace) versus the concordance or Braden scores (red trace) for patients developing in‐house PI throughout their SNF stay.

The results indicated that certain components of the Braden score, specifically the sensory and moisture subscores, had minimal impact on predicting PI risk. The most significant factors increasing the risk of PIs were identified as a recent decrease in overall Braden score, low scores in the nutrition, friction and activity subscales, and a history of previous PI. While the Braden score shows a moderate level of accuracy in predictions (with a global concordance score of 0.604), the survival model surpasses it, achieving a global concordance score of 0.662. This indicates a 10.4% improvement in predictive accuracy, a statistically significant enhancement (Mood's median test *χ* ^2^ = 16.2, *p* < 0.001). Moreover, the research highlights that over the initial 180 days of patient stays in SNFs, the survival model consistently offers superior predictive reliability across numerous time points. This was determined through statistical analysis, including Student's *t*‐tests with adjustments for multiple comparisons. These findings suggest the survival model, including additional data to the Braden score (e.g., patient wound history, demographics), can enhance the accuracy of PI risk assessment and is a potentially more effective tool for assessing patient outcomes in such healthcare settings, providing a significant advantage over traditional methods like the Braden score alone. Figure 1 illustrates the hazard ratios for the features included in the model, Table 2 presents the results of the Cox Proportional Hazards model summary showing coefficient estimates for factors affecting in‐house PI development. Figure 2 illustrates the global concordance indices of our survival model vs Braden scores.

**TABLE 2:** Cox Proportional Hazards model summary showing coefficient estimates for factors affecting in‐house pressure injury (PI) development.

Covariate	coef	exp(coef)	se(coef)	*p*
Age	0.254	1.29	0.023	<0.05
Gender male	0.0856	1.089	0.00574	<0.05
PI in past 90 days	0.3	1.35	0.0118	<0.05
Braden nutrition	−0.442	0.642	0.0122	<0.05
Braden activity	−0.335	0.714	0.01179	<0.05
Braden mobility	−0.285	0.752	0.0108	<0.05
Braden friction	−0.281	0.755	0.00879	<0.05
Braden score decrease in 21 days	0.542	1.72	0.00875	<0.05

Male patients were indicated to be 9% more likely to develop PI in a SNF than female patients which is consistent with previous studies on this patient population.^16^

Results enclosed within the yellow dotted box demonstrate statistical significance across all 10 training folds. Thus, our model exhibits substantial concordance at most evaluated time points. Notable exceptions include the initial phase (approximately up to day 10) and sporadic intervals in the later stages of observation when the wounds are more mature. These deviations may be attributed to fluctuations in the confidence intervals towards the end of the time series. It is important to note that the *p*‐values have been adjusted using the Bonferroni correction for approximately 180 tests (corresponding to one test per time slice) based on an initial alpha level of 0.05. This adjustment results in a particularly stringent threshold for statistical significance.

## 4 DISCUSSION

The study provides a comprehensive analysis of the potential benefits of a refined Braden score, incorporating elements from patient history and specific Braden score components, to improve the prediction accuracy of PI risk in SNF. Our findings contribute towards the growing literature indicating the potential value of data‐driven solutions to PI risk assessment.^12^, ^17^

### 4.1 Enhancement of predictive accuracy

The integration of a survival model trained on a large database of patient stays in SNFs marks a significant advancement in assessing PI risk. By refining the Braden score to include wound‐focused histories and analysing the dynamic changes in Braden scores over time, our study demonstrated a 10.4% improvement in prediction accuracy over traditional methods. We found that patients with a recent drop in Braden scores were 1.72 times more likely to develop PI than patients with stable or increasing scores, underscoring the importance of monitoring Braden scores longitudinally as a highly predictive indicator of imminent PI risk. Notably, a drop in Braden score within the past 21 days (see Figure 1) had the highest positive hazard ratio (HR), indicating this factor is strongly associated with the development of PI. The utility of the Braden score could be enhanced with clinically validated thresholds for deterioration. Clinical decision support in EMRs could identify patients that are imminently at‐risk, developing a PI or adjusting after the heightened period of risk passes. This finding highlights the importance of time‐series analysis, specifically considering the trajectory of the risk score trend. Clinicians may find this challenging if historical data, or the 21‐day history, is being stored or presented in a manner, which is not conducive to identifying trends, that is, using paper‐based documentation methods. It also highlights the importance of regular reassessment and preventative interventions after a decrease in Braden score, as patients are at heightened risk of PI during the subsequent 21‐day period.

Furthermore, our analysis revealed that most, though not all, of the Braden subscales provide valuable information for predicting PI risk. Contrary to previous assumptions inherent in the Braden calculation, not all subscores are weighted equally. Nutrition (HR = −0.44) subscores emerged as the most influential, followed by those for friction (HR = −0.28), activity (HR = −34) and mobility (HR = −0.29). These findings suggest that a nuanced approach to weighing Braden subscales (e.g., nutrition, friction, activity, mobility) could enhance predictive accuracy significantly.

### 4.2 Practicality and implementation

While the study's findings are promising, it is not intended to suggest removal of individual subscores. Rather these findings can be leveraged to improve population risk monitoring and alerting for increasing risk of PI requires careful consideration of practicality. The implementation success will depend on the availability of technological infrastructure, specifically a medical record system capable of undertaking risk modelling based on this survival model, healthcare professionals' training and the adaptability of current practices to incorporate new risk assessment tools. Additionally, the financial implications of adopting such models must be evaluated to ensure the benefits outweigh the costs associated with technological investments and process modifications. Technology that reduces PI prevalence can lower costs by reducing the prevalence and associated costs of care (e.g., clinician time, dressing costs, care reimbursement), regulatory penalties for failing to meet standards of care and litigation arising from newly acquired wounds.^18^, ^19^ Ongoing model performance evaluation could be done to provide formative evaluation and also be warranted to ensure it remains dynamic and responsive to changing risk factors influencing PI outcomes across different clinical contexts. This study opens several avenues for further research. Investigating the applicability of personalized risk assessment models in other patient care settings, such as hospitals or home care, could broaden the impact of this approach. Moreover, longitudinal studies assessing the clinical outcomes of implementing these refined risk assessment tools or alerts would provide valuable insights into their efficacy in reducing the incidence and severity of PIs. Comparative studies using control groups that had access to enhanced PI risk analysis and those using the standard Braden score to evaluate differences in outcomes (e.g., clinical, operational, financial). By combining quantitative data with individualized patient histories, PI risk assessment could be improved leading to more accurate and personalized care strategies. This study therefore reaffirms the importance of leveraging big data and digitalization strategies to improve patient outcomes, highlighting the potential of big data to transform healthcare practices and improve current standardized wound risk scores.

### 4.3 Limitations and interpretation

While the study provides new insights into quantitative risk assessment, it does not evaluate the impact of this model on clinical outcomes directly. Future studies are necessary to determine how the implementation of this risk assessment model affects the development of PIs and their severity in practice. Furthermore, while the findings are promising, the generalizability of the results across different healthcare settings and populations should be approached with caution. Adding to this complexity, patient demographics were found to influence PI risk, with male patients about 9% more likely to develop PI in a SNF than female patients. The risk of developing a PI in‐house increases with patient age, and a history of recent PI is associated with increased risk of new PI. Interestingly, Moisture and Sensory perception scores were not associated with PI risk, suggesting that critical values in these subscores might trigger preventative care that precludes PI. This may mean that these factors may be significant in understanding risk of PI but may be responded to clinically rendering these subscales statistically insignificant in our model despite their potential clinical relevance. It is also important to note the heterogeneity of patients within SNF which may also limit the generalizability of the analysis of risks from out study. It is important to consider that patients should be evaluated holistically in clinical practice.

The risks identified in this study warrant further investigation to understand the underlying mechanisms fully. These additional insights underscore the complexity of PI risk assessment and the potential of a refined Braden score to significantly improve predictive accuracy. As the healthcare industry moves towards more personalized and data‐driven approaches, integrating nuanced models like the one proposed here will be crucial in enhancing patient care and outcomes in SNFs and beyond.

## 5 CONCLUSION

This study advances PI risk assessment in SNFs by refining the Braden score with a survival model trained on the Swift database. This approach addresses the limitations of existing tools, notably their low sensitivity and static nature, by incorporating detailed patient histories and specific score components. Our findings reveal a 10.4% improvement in prediction accuracy, highlighting the potential for more effective, targeted preventative measures against PIs. This challenges the current trend towards subjective clinical judgement, advocating for a data‐driven, personalized strategy in PI risk assessment. By leveraging technological advancements and detailed patient data, this study underscores the importance of precision in healthcare, optimizing resource allocation and improving patient outcomes. However, it is crucial to note that while our model enhances predictive accuracy, its impact on clinical outcomes and practical implementation requires further investigation. Future research should explore the model's applicability across various healthcare settings and its integration into clinical workflows.

This study offers promising evidence towards improving PI prevention strategies in SNFs, advocating for a shift towards quantitative, evidence‐based risk assessment methodologies. This approach not only promises to improve patient care but also contributes to the evolving landscape of healthcare practices in addressing PIs.

## FUNDING INFORMATION

No specific funding provided for this study.

## CONFLICT OF INTEREST STATEMENT

The authors declare no conflicts of interest.

## Data Availability

Research data are not shared.
